# Power gains by using external information in clinical trials are typically not possible when requiring strict type I error control

**DOI:** 10.1002/bimj.201800395

**Published:** 2019-07-02

**Authors:** Annette Kopp‐Schneider, Silvia Calderazzo, Manuel Wiesenfarth

**Affiliations:** ^1^ Division of Biostatistics German Cancer Research Center (DKFZ) Heidelberg Germany

**Keywords:** Bayesian dynamic borrowing of information, evidence synthesis, frequentist error control, historical information, robust prior

## Abstract

In the era of precision medicine, novel designs are developed to deal with flexible clinical trials that incorporate many treatment strategies for multiple diseases in one trial setting. This situation often leads to small sample sizes in disease‐treatment combinations and has fostered the discussion about the benefits of borrowing of external or historical information for decision‐making in these trials. Several methods have been proposed that dynamically discount the amount of information borrowed from historical data based on the conformity between historical and current data. Specifically, Bayesian methods have been recommended and numerous investigations have been performed to characterize the properties of the various borrowing mechanisms with respect to the gain to be expected in the trials. However, there is common understanding that the risk of type I error inflation exists when information is borrowed and many simulation studies are carried out to quantify this effect. To add transparency to the debate, we show that if prior information is conditioned upon and a uniformly most powerful test exists, strict control of type I error implies that no power gain is possible under any mechanism of incorporation of prior information, including dynamic borrowing. The basis of the argument is to consider the test decision function as a function of the current data even when external information is included. We exemplify this finding in the case of a pediatric arm appended to an adult trial and dichotomous outcome for various methods of dynamic borrowing from adult information to the pediatric arm. In conclusion, if use of relevant external data is desired, the requirement of strict type I error control has to be replaced by more appropriate metrics.

## 1 INTRODUCTION

Borrowing of information from an external data source to inform inference in a current trial is gaining popularity in situations where only small samples are available for practical or ethical reasons. In this context, borrowing of information is often also referred to as evidence synthesis or extrapolation, where external data could be historical data or any source of codata. The present work is motivated by a trial in precision medicine in which adults with a specific molecular tumor profile are treated with targeted therapy and response to therapy is assessed. The population of children with this specific molecular profile is too small to warrant a separate pediatric trial. This motivates the implementation of a pediatric stratum in the adult trial and the setting suggests that information from the adult trial should be used for the pediatric stratum as external information. Several approaches have been proposed that dynamically discount the amount of information borrowed from external data based on the discrepancy between the external and current data (also known as prior‐data conflict). These include Bayesian dynamic borrowing methods such as hierarchical models, adaptive power priors, and robust mixture priors, and frequentist approaches such as test‐then‐pool. For comprehensive overviews, see, for example, Viele et al. (2014) and Wadsworth, Hampson, and Jaki (2018). The rationale for using such dynamic borrowing mechanisms is often given by the desire to take into account external information only when it improves inference. However, it seems to be hidden knowledge in the Bayesian community that no power gain is possible when type I error needs to be controlled, which has been stated before by, for example, Psioda and Ibrahim (2018): “If one wishes to control the type I error rate in the traditional frequentist sense, all prior information must be disregarded in the analysis.” These authors also give a formal proof in case of the one‐sample one‐sided test of a normal endpoint in the context of power priors with fixed power parameter, that is, a situation where the same amount of prior information is incorporated independent of the data. Similarly, Grieve (2016), again in the context of constant borrowing of information, acknowledges, referring to FDA and CDRH (2010): “[...] requiring strict control of the type I error results in 100% discounting of the prior information. [...] This [...] is important in the context of the remark in the FDA's Bayesian guidance that ‘it may be appropriate to control the type I error at a less stringent level than when no prior information is used’. I would argue that the FDA's remark is recognition of this phenomenon and an endorsement of a less strict control of type I error [...],” see also Pennello and Thompson (2007) for additional insight. Interest in comparison of operating characteristics of dynamic borrowing approaches has led to several recent comprehensive simulation studies on possible gains with respect to power (see, e.g., Cuffe, 2011; Dejardin et al., 2018; Gamalo‐Siebers et al., 2017; van Rosmalen, Dejardin, van Norden, Löwenberg, & Lesaffre, 2018), but there appears to be no definite answer.

The aim of our study is to clarify why borrowing of information cannot lead to an increased power while strictly controlling type I error. This can be, maybe even somewhat trivially, proven by resorting to the framework of uniformly most powerful (UMP) testing. The calibration of Bayesian procedures, that is, the reliability of Bayesian probability statements under repeated sampling, has been previously investigated: we refer, for example, to Rubin (1984) for a discussion on posterior intervals coverage; moreover, for example, Lehmann (1986) and Berger (1985) provide a decision‐theoretic view on the relationship between frequentist and Bayesian test decisions. However, an easily accessible reference addressing the incorporation of historical information in the context of UMP testing seems to be missing. In case of borrowing of information by a constant amount, the finding may be not very surprising. However, it may feel counterintuitive in case of dynamic borrowing of information. Inclusion of prior information may always be understood as adding additional samples, for example, from a historical trial. Dynamic borrowing of information aims at adapting the number of added external samples depending on the discrepancy between external and current data. Thus, intuition may suggest that power may be increased (where prior information and the true parameter value generating the current data are close), while still controlling type I error (where prior and current true parameter are disparate). However, as it will be shown, the apparent advantage vanishes when accounting for the sampling variability of the current data (while external data have been obtained in the past and hence is fixed). The result primarily follows from clarifying that the decision criterion of any decision rule that borrows external information can be viewed in terms of a decision rule that only depends on the current data, whereas the external information is fixed upfront.

We state our finding in very general terms in Section 2. To describe it in the Bayesian context, we show a general reformulation in Subsection 2.2. In Section 3, we show that our argument holds for typical situations encountered in clinical trials settings. In Section 4, we use as a very simple situation a one‐sided comparison in a one‐arm trial evaluating a dichotomous endpoint and investigate a number of Bayesian and also a frequentist method for borrowing information to illustrate the general proof. To conclude, we discuss in Section 5 the implications and justifications under which circumstances borrowing of information can be beneficial.

## 2 BORROWING OF INFORMATION WHEN A UNIFORMLY MOST POWERFUL TEST EXISTS

### 2.1 General framework

We first consider the general scenario in which a trial is performed to evaluate an endpoint and a UMP test exists. Assume that the endpoint has probability density function $f_{\theta }\left(x\right)$ and the hypotheses investigated in the trial is one‐sided, without loss of generality,

$$H_{0}:\theta \leq \theta_{0}\;\text{versus}\;H_{1}:\theta >\theta_{0}.$$

Let $D_{1}=\left\{X_{1},\ldots ,X_{n_{1}}\right\}$ be the random variables from which the observations of the current trial, $d_{1}=\left\{x_{1},\ldots ,x_{n_{1}}\right\}$, are obtained. Note that capital letters indicate random variables, whereas small letters indicate the observations from these random variables. If the trial is evaluated, the test decision will be performed by the UMP test

$$\varphi_{\text{UMP}}\left(d_{1}\right)=\left\{\begin{array}{cc} 1 & \text{if}\;T\left(d_{1}\right)>t_{0},\\ \gamma & \text{if}\;T\left(d_{1}\right)=t_{0},\\ 0 & \text{if}\;T\left(d_{1}\right)<t_{0}, \end{array}\right.$$

where $T\left(x_{1},\ldots ,x_{n_{1}}\right)=T\left(d_{1}\right)$ is a sufficient test statistic for $f_{\theta }\left(x\right)$ and *t*_0_ is chosen such that $E_{\theta_{0}}\left[\varphi_{\text{UMP}}\left(T\left(D_{1}\right)\right)\right]=\alpha$, that is, the test controls (frequentist) type I error, where α denotes the significance level of the test. For a given *d*_1_, the value of the test function $\varphi_{\text{UMP}}\left(d_{1}\right)$ corresponds to the probability to reject *H*_0_ given *d*_1_ is observed. On the boundary $T\left(d_{1}\right)=t_{0}$, the decision is randomized with probability γ. For continuous distributions, this has no practical implication, but for discrete distributions, randomization is unacceptable in practice. Hence, we will adopt the convention to set γ to 0, which implies that the level of the test may not fully attain the nominal significance level, that is, $E_{\theta_{0}}\left[\varphi_{\text{UMP}}\left(T\left(D_{1}\right)\right)\right]\leq \alpha$. Thus, the UMP test can be written as

(1) $$\varphi_{\text{UMP}}\left(d_{1}\right)=\left\{\begin{array}{cc} 1 & \text{if}\;T(d_{1})\in C\\ 0 & \text{if}\;T(d_{1})\notin C \end{array}\right.$$

with the set $C=(t_{0},\infty )$ in this one‐sided test.

Now assume that external information *d*_0_ is available, which is independent of *d*_1_, and should be used to inform the test decision for the current trial. This external information *d*_0_ is not random but fixed, and a decision rule is formulated based on the observed results of the current trial, *d*_1_, that will again depend on the result of the sufficient test statistic $T(d_{1})$. Incorporation of the external information, *d*_0_, is achieved by modifying the critical region of the decision rule, $C_{d_{0}}$, according to *d*_0_. Hence, a test function $\varphi_{B}$ is identified such that

(2) $$\varphi_{B}\left(d_{1};d_{0}\right)=\left\{\begin{array}{cc} 1 & \text{if}\;T\left(d_{1}\right)\in C_{d_{0}}\\ 0 & \text{if}\;T\left(d_{1}\right)\notin C_{d_{0}}. \end{array}\right.$$

As the external information *d*_0_ is fixed, $\varphi_{B}\left(.,d_{0}\right)$ is, in fact, a function only of the current data *d*_1_.

If strict type I error control is required, then $C_{d_{0}}$ will be selected as the largest set $C_{d_{0}}$ such that $E_{\theta_{0}}\left[\varphi_{B}\left(D_{1};d_{0}\right)\right]=E_{\theta_{0}}\left[\varphi_{B}\left(D_{1};D_{0}\right)|D_{0}=d_{0}\right]\leq \alpha$. Note that for continuous distributions, $C_{d_{0}}$ is selected such that α will be reached, but for discrete distributions, the significance level may not be attained. As the UMP test for *d*_1_, $\varphi_{\text{UMP}}$, exists, the power of $\varphi_{B}$ cannot exceed that of the UMP test, that is, $E_{\theta }\left[\varphi_{B}\left(D_{1};d_{0}\right)\right]\leq E_{\theta }\left[\varphi_{\text{UMP}}\left(D_{1}\right)\right]$ for all $\theta >\theta_{0}$. This shows that no power gain can be expected from borrowing of external information when strict control of type I error rate is required. This argument is true for any borrowing mechanism, even when borrowing from external information is discounted in case of conflict between external and current data.

Note that the key point of the argument is the difference between the conditional expectation $E_{\theta }\left[\varphi_{B}\left(D_{1};D_{0}\right)|D_{0}=d_{0}\right]$ and the unconditional expectation $E_{\theta }\left[\varphi_{B}\left(D_{1};D_{0}\right)\right]$. If the external information was random as well and if it was generated from the same distribution as the current data, then a power gain can be achieved even when strict control of type I error rate is required. However, this is not a situation that generally occurs in practice because it would mean that *D*_1_ and *D*_0_ are evaluated in a pooled analysis as coming from the same trial.

### 2.2 Bayesian borrowing of information

The formulation of the test function $\varphi_{B}$ in (2), and specifically of the critical region $C_{d_{0}}$ is very general. If borrowing of external information is achieved by Bayesian methods, the decision function $\varphi_{B}\left(D_{1};d_{0}\right)$ for the one‐sided test is given by

(3) $$\varphi_{B}\left(d_{1};d_{0}\right)=\left\{\begin{array}{cc} 1 & \text{if}\;P\left(\theta >\theta_{0}|d_{1};d_{0}\right)>c_{d_{0}}\\ 0 & \text{if}\;P\left(\theta >\theta_{0}|d_{1};d_{0}\right)\leq c_{d_{0}}, \end{array}\right.$$

where the posterior is induced by a prior that incorporates the external information *d*_0_ (this is indicated here by “$P(.|.;d_{0})$”), and $c_{d_{0}}\in \left[0,1\right)$. The posterior can be viewed as a function of the sufficient statistics, $P\left(\theta >\theta_{0}|d_{1};d_{0}\right)=g_{d_{0}}\left(T\left(d_{1}\right)\right)$ (see, e.g., Sahu & Smith, 2006). If the function $g_{d_{0}}$ is strictly monotone, $c_{d_{0}}$ can be determined such that $g_{d_{0}}^{-1}\left(c_{d_{0}}\right)=t_{0}$ in (1). Hence, $g_{d_{0}}\left(T\left(d_{1}\right)\right)>c_{d_{0}}$ corresponds to the condition $T\left(d_{1}\right)\in C_{d_{0}}$ in (2), and therefore, $\varphi_{B}$ and $\varphi_{\text{UMP}}$ coincide. Strict monotonicity can, however, often not been shown in general. If $g_{d_{0}}$ is not strictly monotone, then it may occur that no $c_{d_{0}}$ can be determined such that $\varphi_{B}=\varphi_{\text{UMP}}$. In this case, either $\varphi_{B}$ does not control type I error, that is, there is $\theta \leq \theta_{0}$ with $E_{\theta }\left[\varphi_{B}\left(T\left(D_{1}\right)\right)\right]>\alpha$, or it does control type I error but there exists $\theta >\theta_{0}$ with $E_{\theta }\left[\varphi_{B}\left(D_{1};d_{0}\right)\right]<E_{\theta }\left[\varphi_{\text{UMP}}\left(D_{1}\right)\right]$. In Section 4, the selection of $C_{d_{0}}$ will be illustrated for different methods of borrowing of external information. For Bayesian borrowing methods, monotonicity of $g_{d_{0}}$ will be discussed and, where appropriate, $c_{d_{0}}$ will be determined such that $\varphi_{B}$ and $\varphi_{\text{UMP}}$ coincide.

## 3 EXAMPLES OF SITUATIONS IN WHICH UMP TESTS EXISTS

For extensive and comprehensive discussions about testing problems for which UMP tests exist, see Lehmann (1986). Here, we report a few situations in which UMP tests exist that we consider most relevant for the application in clinical trials. A general requirement is that the endpoint has probability density function $f_{\theta }\left(x\right)$ with monotone likelihood ratio in the sufficient statistics T(x). This is valid if the endpoint belongs to a one‐parameter exponential family, that is, its probability density function has the form

$$f_{\theta }\left(x\right)=g\left(\theta \right)h\left(x\right)\exp\left(\eta \left(\theta \right)T\left(x\right)\right),$$

and if η(θ) is strictly monotone. The most common distributions such as normal with known variance and binomial with fixed number of trials are one‐parameter exponential families, but so do also the exponential, Poisson, as well as various much less common clinical trial outcomes.

For one‐group one‐sided hypotheses, but also for certain two‐sided hypotheses of the form $H_{0}:\theta \leq \theta_{1}\;\text{or}\;\theta \geq \theta_{2}$ with $\theta_{1}<\theta_{2}$ versus $H_{1}:\theta_{1}<\theta <\theta_{2}$, Lehmann (1986) shows that UMP tests exist. Such two‐sided hypotheses are important to show the equivalence of treatments in the context of clinical trials, that is, to show that two treatments are not too different in characteristics. The UMP tests for these two‐sided hypotheses can be formulated in the same general formula (1) with critical region C appropriately adjusted. For certain two‐group one‐sided comparisons, UMP tests exist as well, for example, for comparison of two means of normal distributions with identical and known variance, that is, the two‐sample normal test.

For two‐sided hypotheses of the type $H_{0}:\theta_{1}\leq \theta \leq \theta_{2}$ versus $H_{1}:\theta \leq \theta_{1}\;\text{or}\;\theta \geq \theta_{2}$ or the hypothesis $H_{0}:\theta =\theta_{0}$ versus $H_{1}:\theta \neq \theta_{0}$, UMP tests do not exist in general. In these situations, however, often UMP‐unbiased tests exist and have the form (1) with appropriately adjusted rejection region C, that is, this test is UMP among unbiased tests. Unbiasedness requires that $E_{\theta }\left[\varphi_{B}\left(D_{1};d_{0}\right)\right]\geq \alpha$ for θ from the alternative and that type I error is controlled, that is, $E_{\theta_{0}}\left[\varphi_{B}\left(D_{1};d_{0}\right)\right]\leq \alpha$ for θ from the null hypothesis. Requiring that the null hypothesis is more easily rejected when it is false than when it is true seems, however, a reasonable condition and should be fulfilled in practice. The argument given in Section 2 therefore can be extended to situations in which UMP do not, but UMP‐unbiased tests exist. Important situations for which UMP unbiased tests exist also include the (one‐sided or two‐sided) comparison of two groups of Poisson and binomial variables (again see Lehmann, 1986).

## 4 EXAMPLE: ONE‐ARM TRIAL WITH DICHOTOMOUS ENDPOINT

An intuitive exemplification of the general result shown in Section 2 is provided in the following. We consider the design of a pediatric single‐arm phase II trial with binary outcome, for example, response to treatment. The response rate considered as uninteresting is the response rate observed in earlier trials, *p*_0_ ($=\theta_{0}$ in the notation of Section 2). The aim of the trial is to reach or exceed a target level of response larger than *p*_0_. Assume that information about the effect of the identical treatment is available from a trial performed in adults. Literature suggests that external information can be used to increase power, particularly if the amount of borrowing is adapted to the conformity of current and external information.

### 4.1 Planning the pediatric arm with stand‐alone evaluation

The number of responders $R_{\text{ped}}$ in the pediatric trial of size $n_{\text{ped}}$ follows a binomial distribution with response rate $p_{\text{ped}}$:

$$R_{\text{ped}}|p_{\text{ped}}∼\text{Bin}\left(n_{\text{ped}},p_{\text{ped}}\right).$$

As a stand‐alone trial, the pediatric trial is designed to test $H_{0}:p_{\text{ped}}\leq p_{0}$ against the alternative $H_{1}:p_{\text{ped}}>p_{0}$, controlling the significance level by α, for example, α=.05. We consider here a simple single‐stage design and present it in a frequentist and in a Bayesian approach. For illustration purposes, the number of pediatric patients in the trial is assumed to be $n_{\text{ped}}=40$ and the null hypothesis value is assumed to be $p_{0}=.2$.

#### 4.1.1 Frequentist design of the stand‐alone pediatric trial

As the binomial distribution with fixed number of trials is a one‐parametric exponential family, a UMP level α test exists. For $n_{\text{ped}}=40$ and α=.05 the test decision is given by

(4) $$\varphi_{\text{UMP}}\left(r_{\text{ped}}\right)=\left\{\begin{array}{cc} 1 & \text{if}\;r_{\text{ped}}>12,\;\text{or,}\;\text{equivalently,}\;r_{\text{ped}}\geq 13\\ 0 & \text{if}\;r_{\text{ped}}\leq 12. \end{array}\right.$$

Due to the discreteness of the distribution, the significance level of .05 is not attained, and the actual type I error rate is α=.043.

#### 4.1.2 Bayesian design of the stand‐alone pediatric trial

In the Bayesian framework, we assume a beta distribution as prior of the response rate $p_{\text{ped}}$:

$$\pi \left(p_{\text{ped}}\right)=\text{Be}\left(s_{1},s_{2}\right)\;\text{with}\;s_{1},s_{2}>0,$$

choosing, for example, Jeffrey's prior with $s_{1}=s_{2}=.5$.

Several options exist for the evaluation of treatment efficacy and we consider here a decision rule based on the posterior distribution of response probability: *H*_0_ will be rejected if

(5) $$P(p_{\text{ped}}>p_{0}|r_{\text{ped}},n_{\text{ped}})\geq c.$$

The critical boundary *c* is chosen such that the desired type I error rate is controlled. In this specific situation, selection of c=.95 ensures that type I error is controlled by α=5% (see, e.g., Kopp‐Schneider et al., 2019).

The decision rule on the basis of posterior probability (5) can be converted to a decision rule on the basis of number of responders $r_{\text{ped}}$ among $n_{\text{ped}}$ treated children by use of what was called a “boundary function” in Kopp‐Schneider et al. (2019). This is achieved by checking for every potential outcome $r_{\text{ped}}$ whether $P(p_{\text{ped}}>p_{0}|r_{\text{ped}},n_{\text{ped}})$ exceeds *c*. The smallest integer for which this is the case is the critical number *b* and *H*_0_ will be rejected if

(6) $$r_{\text{ped}}\geq b.$$

**Figure 1:**
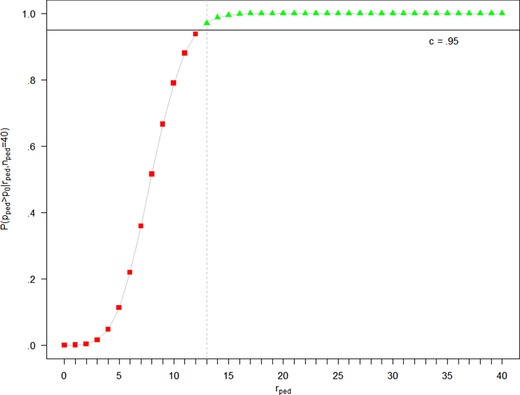
Posterior probability $P(p_{\text{ped}}>p_{0}|r_{\text{ped}},n_{\text{ped}})$ as a function of the number of responders $r_{\text{ped}}$

In case of $n_{\text{ped}}=40$ and c=.95, the critical number of responders to reject *H*_0_ is b=13. Hence, the Bayesian decision rule based on (5) is identical to the frequentist decision rule for the UMP test given in (4). The correspondence between the decision rule in terms of posterior probability and in terms of number of responders is illustrated by showing the posterior probability $P(p_{\text{ped}}>p_{0}|r_{\text{ped}},n_{\text{ped}})$ as a function of the number of responders $r_{\text{ped}}$ in Figure 1.

The posterior probability is a monotonically increasing function of the number of responders (see Kopp‐Schneider et al., 2019), irrespective of the specific beta prior distribution. For this reason, the correspondence of the decision rule in terms of posterior probability and the decision rule in terms of number of responders holds in general for reasonable *c* that can be reached for the specific prior distribution (note that $P(p_{\text{ped}}>p_{0}|r_{\text{ped}}=n_{\text{ped}})$ may be smaller than 1 for an informative prior with large mass below *p*_0_). This correspondence is given by:

For every critical boundary $c\in [0,P\left(p_{\text{ped}}>p_{0}|r_{\text{ped}}=n_{\text{ped}}\right)]$, there exists a unique critical number $b\in \{0,1,\ldots ,n_{\text{ped}}\}$ with

(7) $$P\left(p_{\text{ped}}>p_{0}|r_{\text{ped}},n_{\text{ped}}\right)\geq c\Leftrightarrow r_{\text{ped}}\geq b.$$

Figure 1 shows that the rejection region can be either read from the *x*‐ or the *y*‐axis. The power function for the test decision can hence be written in two equivalent ways:

(8) $$\begin{array}{cc} \text{Power} & =f\left(p_{\text{true}}\right)=E_{p_{\text{true}}}\left[\varphi_{\text{UMP}}\left(r_{\text{ped}}\right)\right]\\ & =\sum_{r_{\text{ped}}=0}^{n_{\text{ped}}}P\left(R_{\text{ped}}=r_{\text{ped}}|p_{\text{true}}\right)1_{\left\{r_{\text{ped}}\geq b\right\}}\\ & =\sum_{r_{\text{ped}}=0}^{n_{\text{ped}}}P\left(R_{\text{ped}}=r_{\text{ped}}|p_{\text{true}}\right)1_{\left\{P\left(p_{\text{ped}}>p_{0}|r_{\text{ped}},n_{\text{ped}}\right)\geq c\right\}}. \end{array}$$

**Figure 2:**
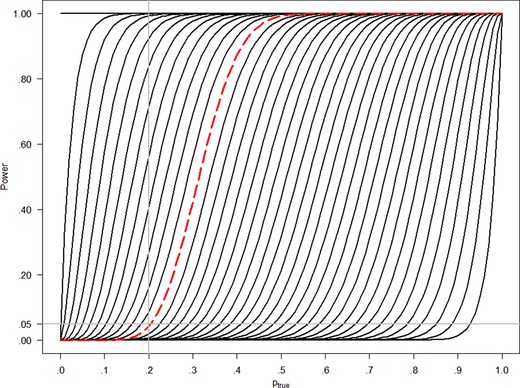
All possible power functions for UMP‐tests in the situation $n_{\text{ped}}=40$, varying the threshold *b* for the observed number of responders, or equivalently the threshold *c* for the posterior probability. The values of $p_{\text{true}}=.2$ and α=.05 are indicated as vertical and horizontal gray lines and the power curve for b=13, corresponding to the 5% level UMP test, is indicated in dashed red

For every threshold in terms of number of responders, *b*, there exists one power function. All possible power functions for UMP tests in $n_{\text{ped}}=40$ with varying type I error rate α, and correspondingly varying threshold *b* or *c*, are shown in Figure 2, with the power function for b=13 highlighted as dashed red line.

### 4.2 Planning the pediatric arm with borrowing from external information

Let us assume that information is available from a trial in $n_{\text{adu}}$ adults in a very similar clinical situation, that is, with patients with the same disease and the same treatment, and assume that $r_{\text{adu}}$ responders were observed in this trial. Thus, $R_{\text{adu}}|p_{\text{adu}}∼\text{Bin}\left(n_{\text{adu}},p_{\text{adu}}\right)$ and in terminology of Section 2, $d_{0}=\left\{x_{1},\ldots ,x_{n_{\text{adu}}}\right\}$ with $T\left(d_{0}\right)=r_{\text{adu}}$. Note, however, that realizations $r_{\text{adu}}$ of $R_{\text{adu}}$ are observed and fixed before the pediatric trial. For simplicity, abbreviate $d_{0}=\left\{r_{\text{adu}};n_{\text{adu}}\right\}$. Information from the adult trial is borrowed with the hope to increase the power of the pediatric trial. Many approaches are available for including the external (adult) information; for reviews, see, for example, Viele et al. (2014) and Rosmalen et al. (2018). A natural way to include the external information is to use a Bayesian design for the pediatric trial and replace the weakly informative prior $\pi (p_{\text{ped}})$ by an informative prior obtained as posterior distribution from the adult trial, that is, $\pi \left(p_{\text{ped}}\right)=\pi \left(p_{\text{ped}}|d_{0}\right)$ and hence use $P(p_{\text{ped}}>p_{0}|r_{\text{ped}},n_{\text{ped}};r_{\text{adu}},n_{\text{adu}})$ for decision. Note that $P(p_{\text{ped}}>p_{0}|r_{\text{ped}},n_{\text{ped}};r_{\text{adu}},n_{\text{adu}})$ equals $g_{d_{0}}\left(T\left(d_{1}\right)\right)$ as introduced in Section 2.2. For the rest of this section, we assume that the adult trial was performed with 40 patients of which 12 responded to treatment, that is, $d_{0}=\left\{12;40\right\}$, corresponding to an observed response rate of ${\hat{p}}_{\text{adu}}=.3$ and a posterior mean of 12.5/41=.305 induced by Jeffrey's prior.

#### 4.2.1 Borrowing from the adult trial using the power prior approach

In the power prior approach, the prior for the pediatric trial is proportional to the likelihood of the external data $L(p;d_{0})$ raised to the power of a weight parameter δ∈[0,1], multiplied by the initial prior

$$\pi \left(p|d_{0},\delta \right)\propto L{\left(p;d_{0}\right)}^{\delta }\pi \left(p\right).$$

The weight parameter determines how much of the external information is incorporated. Extreme cases are δ=0, when information from *d*_0_ is discarded and δ=1, when *d*_0_ is completely taken into account. For developments of the power prior approach, see, for example, Duan, Ye, and Smith (2006); Gravestock and Held (2017); Ibrahim and Chen (2000); Ibrahim, Chen, Gwon, and Chen (2015); Neuenschwander, Branson, and Spiegelhalter (2009) and Nikolakopoulos, Tweel, and Roes (2017).

##### Fixed power parameter

Incorporating the adult data *d*_0_ with a fixed power parameter δ is equivalent to using an updated (beta) prior for the response rate in the pediatric arm. The prior is hence $\pi \left(p_{\text{ped}}|d_{0},\delta \right)=\text{Be}\left(a+\delta r_{\text{adu}},b+\delta \left(n_{\text{adu}}-r_{\text{adu}}\right)\right)$. With a choice of, for example, δ=.5 the posterior probability $P(p_{\text{ped}}>p_{0}|r_{\text{ped}},n_{\text{ped}};r_{\text{adu}},n_{\text{adu}})$ is shown in Figure 4 as a function of the number of pediatric responders $r_{\text{ped}}$. Since ${\hat{p}}_{\text{adu}}=.3>p_{0}=.2$, the posterior probability with borrowing from adults is larger than the posterior probability without borrowing from adults for all $r_{\text{ped}}$. In this situation, $P(p_{\text{ped}}>p_{0}|r_{\text{ped}},n_{\text{ped}};r_{\text{adu}},n_{\text{adu}})>c=.95$ is reached for $r_{\text{ped}}\geq 12$, see Table 1. As shown in Kopp‐Schneider et al. (2019), $P(p_{\text{ped}}>p_{0}|\text{Data})$ is monotonically increasing for any beta prior distribution, that is, $g_{d_{0}}\left(T\left(d_{1}\right)\right)=P\left(p_{\text{ped}}>p_{0}|r_{\text{ped}},n_{\text{ped}};r_{\text{adu}},n_{\text{adu}}\right)$ is monotonically increasing in $r_{\text{ped}}$ and the threshold can be adjusted to control type I error: Selecting $c_{d_{0}}=.97$ in the terminology of Section 2.2 leads to $P\left(p_{\text{ped}}>p_{0}|r_{\text{ped}},n_{\text{ped}};r_{\text{adu}},n_{\text{adu}}\right)>c_{d_{0}}=.97$ whenever $r_{\text{ped}}\geq 13$. Hence, if strict type I error control is required, the test function $\varphi_{B}$ is given by

(9) $$\varphi_{B}\left(d_{1};d_{0}\right)=\left\{\begin{array}{cc} 1 & \text{if}\;T\left(d_{1}\right)=r_{\text{ped}}\in C_{d_{0}}=\left\{13,\ldots ,40\right\}\\ 0 & \text{if}\;r_{\text{ped}}\notin \left\{13,\ldots ,40\right\}, \end{array}\right.$$

and is obviously identical to $\varphi_{\text{UMP}}$ in (4).

**Table 1:** Posterior probability $P(p_{\text{ped}}>p_{0}|\text{Data})$ without borrowing and with different borrowing methods for the relevant range of $r_{\text{ped}}$ values. For the power prior, for the robust mixture prior approach and for the hierarchical model, external information was $d_{0}=\left\{12;40\right\}$. For extreme borrowing, external information was $d_{0}^{\prime }=\left\{30;100\right\}$

$r_{\text{ped}}$	9	10	11	12	13	14	15	16
Without borrowing	0.6657	0.7898	0.8799	0.9377	0.9707	0.9875	0.9951	0.9983
Fixed power parameter, δ=.5	0.8344	0.8987	0.9421	0.9690	0.9845	0.9928	0.9968	0.9987
EB power parameter	0.9156	0.9490	0.9708	0.9841	0.9918	0.9960	0.9981	0.9992
Robust mixture prior, w=0.5	0.8678	0.9225	0.9568	0.9772	0.9886	0.9946	0.9976	0.9990
Hierarchical model	0.7748	0.8624	0.9225	0.9585	0.9795	0.9910	0.9961	0.9986
Extreme borrowing	0.6657	0.7898	0.8799	0.9977	0.9707	0.9875	0.9951	0.9983

##### Adaptive power parameter

**Figure 3:**
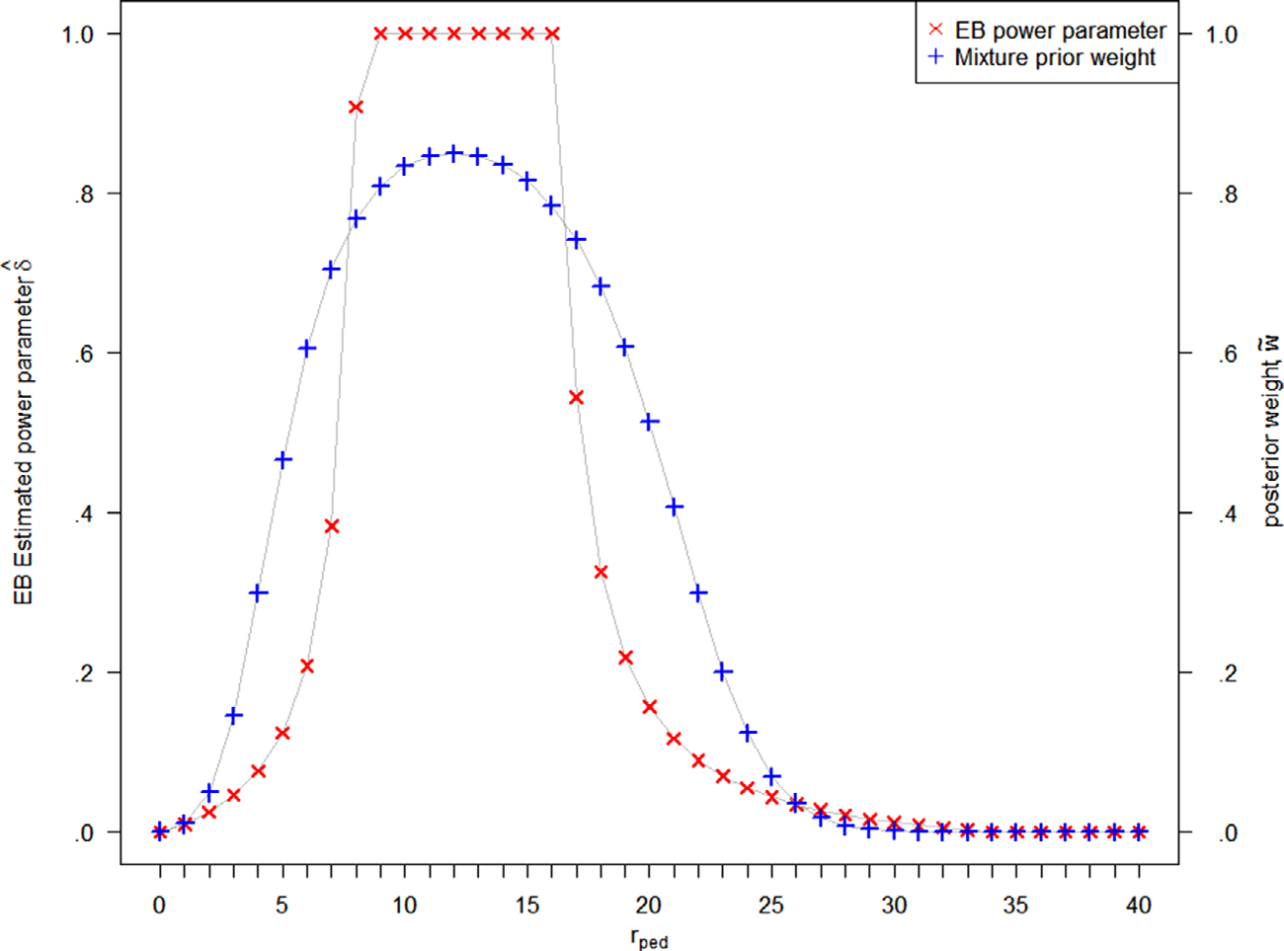
Adaptive power parameter $\hat{\delta }$ determined by empirical Bayes and posterior weight of the robust mixture prior. Results are given for $n_{\text{ped}}=40$, adult information $d_{0}=\left\{r_{\text{adu}}=12;n_{\text{adu}}=40\right\}$, and prior weight w=0.5 for the robust mixture prior approach

In the adaptive power prior approach, the power prior parameter δ depends on the similarity of the current and the external data, such that δ is large when the adult and pediatric data are similar and small if they are conflicting. We follow Gravestock and Held (2017) who propose to use an empirical Bayes (EB) approach for estimation of $\delta (r_{\text{ped}},n_{\text{ped}};r_{\text{adu}},n_{\text{adu}})$ that maximizes the marginal likelihood of δ. Figure 3 shows the resulting values of $\hat{\delta }\left(r_{\text{ped}},n_{\text{ped}}=40;r_{\text{adu}}=12,n_{\text{adu}}=40\right)$ for varying $r_{\text{ped}}$. Full borrowing of the adult information is achieved when the observed pediatric response rate is close to the adult response rate of .3, that is, for 9 to 16 pediatric responders.

**Figure 4:**
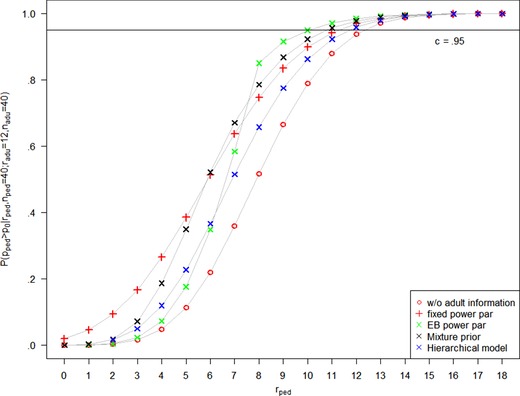
Posterior probability $P(p_{\text{ped}}>p_{0}|\text{Data})$ as a function of the number of responders $r_{\text{ped}}$ without external information, and with adult information $d_{0}=\left\{r_{\text{adu}}=12,n_{\text{adu}}=40\right\}$, using a fixed power parameter (δ=.5), the EB power parameter, a mixture prior approach with w=0.5, and a hierarchical model. The posterior probability for extreme borrowing follows the one without external information, except for $r_{\text{ped}}=12$, where it jumps to 0.9977

The plot of the posterior probability in Figure 4 nicely reflects the discounting of external information for conflicting current and external data. The threshold $P(p_{\text{ped}}>p_{0}|r_{\text{ped}},n_{\text{ped}};r_{\text{adu}},n_{\text{adu}})>c=.95$ is reached for $r_{\text{ped}}\geq 11$, see Table 1. In case of dynamic borrowing, the posterior probability $P(p_{\text{ped}}>p_{0}|r_{\text{ped}},n_{\text{ped}};r_{\text{adu}},n_{\text{adu}})$ is not necessarily a monotonically increasing function of $r_{\text{ped}}$. However, in the case considered here, Table 1 and Figure 4 show that it is monotonically increasing in the relevant range of $r_{\text{ped}}$. Adjustment of the threshold to, for example, $c_{d_{0}}=.99$ leads to the rejection region $r_{\text{ped}}\geq 13$, that is, $C_{d_{0}}=\left\{13,\ldots ,40\right\}$, and $\varphi_{B}$ and $\varphi_{\text{UMP}}$ coincide.

#### 4.2.2 Borrowing from the adult trial using the robust mixture prior approach

Schmidli et al. (2014) among others propose the use of a robust mixture prior as convex combination of an uninformative prior and a prior that incorporates the external information in the form

$$\pi \left(p|d_{0},w\right)=w\text{Be}\left(a_{H},b_{H}\right)+\left(1-w\right)\text{Be}\left(a_{U},b_{U}\right),$$

where $a_{H}=.5+r_{\text{adu}}$ and $b_{H}=.5+n_{\text{adu}}-r_{\text{adu}}$ correspond to the posterior from the adult trial and $a_{U}=.5$ and $b_{U}=.5$ correspond to Jeffrey's prior. The posterior in this approach is a convex combination of two beta distributions with weight

$$\tilde{w}=\frac{B(a_{H}+r_{\text{ped}},b_{H}+n_{\text{ped}}-r_{\text{ped}})}{B(a_{H},b_{H})}\frac{w}{c},$$

where $c=wB\left(a_{H}+r_{\text{ped}},b_{H}+n_{\text{ped}}-r_{\text{ped}}\right)/B\left(a_{H},b_{H}\right)+\left(1-w\right)B\left(a_{U}+r_{\text{ped}},b_{U}+n_{\text{ped}}-r_{\text{ped}}\right)/B\left(a_{U},b_{U}\right)$ and B(.,.) denotes the beta function. The posterior weight depends on the similarity of the external and the current data, as shown in Figure 3.

The plot of the posterior probability in Figure 4 shows that borrowing achieved by the robust mixture prior approach is between fixed and EB‐adaptive power prior approach for a prior weight of 0.5. The implications for the decision $P(p_{\text{ped}}>p_{0}|r_{\text{ped}},n_{\text{ped}};r_{\text{adu}},n_{\text{adu}})>c=.95$ are that the threshold is exceeded for $r_{\text{ped}}\geq 12$. Again, this can be remedied by adjusting the threshold to, for example, $c_{d_{0}}=.98$ (see Table 1), which leads to the rejection region $r_{\text{ped}}\geq 13$, that is, $C_{d_{0}}=\left\{13,\ldots ,40\right\}$ and the test function $\varphi_{B}$ coincides with $\varphi_{\text{UMP}}$ again.

#### 4.2.3 Borrowing from the adult trial using a Bayesian hierarchical model

Dynamic borrowing of information from the adult trial can also be implemented using a hierarchical model. Although a hierarchical model can be specified in the context of a beta‐binomial model, we follow the more common approach and set up a normal hierarchical model for the log‐odds of response probabilities.

Thus, we assume

(10) $$\log\left(\frac{p_{j}}{1-p_{j}}\right)|\mu ,\tau^{2}∼N\left(\mu ,\tau^{2}\right),\;j\in \left\{\text{adu},\text{ped}\right\}.$$

The heterogeneity parameter τ^2^ controls the degree of borrowing: The model reduces to full borrowing (complete pooling) of information from adults to children in case of $\tau^{2}=0$, whereas independent inference with respect to external and current data is given for $\tau^{2}=\infty$. To achieve dynamic borrowing (partial pooling), we assume a half‐normal prior for τ with scale 1, which is a common choice. Further, we assume an improper flat prior for μ.

Note that equivalence of model (10) to Pocock's bias model, commensurate priors, and power priors can be shown for situations with a single external source of information (Neuenschwander, Roychoudhury, & Schmidli, 2016) as well as to a model that is termed a “reference model” by Röver and Friede (2018) when interest is restricted to shrinkage estimates in the pediatric trial. In case of the latter, model (10) is rewritten such that information is only borrowed from adults to children in place of viewing both sources as exchangeable.

The plot of the posterior probability in Figure 4 suggests that the hierarchical model performs unfavorably compared to the other adaptive borrowing methods in the setting considered here. The implication for the decision $P(p_{\text{ped}}>p_{0}|r_{\text{ped}},n_{\text{ped}};r_{\text{adu}},n_{\text{adu}})>c=.95$ is that the threshold is exceeded for $r_{\text{ped}}\geq 12$. Again, this can be remedied by adjusting the threshold to, for example, $c_{d_{0}}=.97$ (see Table 1), which leads to the rejection region $r_{\text{ped}}\geq 13$, that is, $C_{d_{0}}=\left\{13,\ldots ,40\right\}$ and the test function $\varphi_{B}$ again coincides with $\varphi_{\text{UMP}}$.

#### 4.2.4 Borrowing from the adult trial using test‐then‐pool

A frequentist approach to incorporate external information depending on commensurability of data sources is to perform a two‐stage analysis, see, for example, Viele et al. (2014). First, a hypothesis test of equal rates between the current and external data is performed. In the second stage, current data are evaluated separately, that is, without including external data, if the hypothesis in the first stage is rejected. If the hypothesis is not rejected, a pooled analysis is performed in the second stage.

In the current setting, for example, Fisher's exact test is performed to test the first‐stage hypothesis $H_{0}^{b}:p_{\text{ped}}=p_{\text{adu}}\;\text{versus}\;H_{1}^{b}:p_{\text{ped}}\neq p_{\text{adu}}$. Since *d*_0_ is fixed, this corresponds to evaluating the *p*‐value for every constellation of $r_{\text{ped}}$ and $n_{\text{ped}}-r_{\text{ped}}$. Depending on the significance level selected for testing $H_{0}^{b}$, for example, $\alpha^{b}=20\%$, this leads to separate analysis for $r_{\text{ped}}\in \left\{0,\ldots ,6\right\}\cup \left\{19,\ldots ,40\right\}$ and pooled analysis for $r_{\text{ped}}\in \left\{7,\ldots ,18\right\}$.

In the second stage, the significance levels for separate and pooled frequentist analysis are selected. If, for example, α=5% is selected in both cases and keeping in mind that b=13 is the decision boundary for the separate test (see (4)), *H*_0_ will be accepted for $r_{\text{ped}}\in \left\{0,\ldots ,6\right\}$ and rejected for $r_{\text{ped}}\in \left\{19,\ldots ,40\right\}$. In the pooled frequentist analysis, the decision boundary is $b_{\text{pooled}}=23$ responders of a total sample size of $n_{\text{ped}}+n_{\text{adu}}=80$ patients. The number of adult responders contributing to this number is fixed by $r_{\text{adu}}=12$, and hence, the second stage test only depends on $r_{\text{ped}}$. Thus, $r_{\text{ped}}\geq 23-r_{\text{adu}}=11$ would then lead to rejection of *H*_0_. Hence, with a choice of α=5% in the second stage, the overall procedure is associated with type I error inflation.

However, adjustment of the second‐stage test significance level for the pooled analysis to, for example, $\alpha_{d_{0}}=2\%$ would require a decision boundary of $b_{\text{pooled}}=25$, that is, $r_{\text{ped}}\geq 13$. For the separate analysis in the second stage, $\alpha_{d_{0}}=2\%$ requires at least $r_{\text{ped}}\geq 14$ for rejecting *H*_0_. Taking everything together, selection of $\alpha^{b}=20\%$ in the first stage and $\alpha_{d_{0}}=2\%$ in separate and pooled analysis in the second stage leads to rejecting *H*_0_ for $r_{\text{ped}}\geq 13$, that is, $C_{d_{0}}=\left\{13,\ldots ,40\right\}$, hence a procedure with type I error control but again no power gain.

#### 4.2.5 Borrowing from the adult trial using “extreme borrowing”

To show the effect of nonmonotonicity of $g_{d_{0}}$ for our argument, an “extreme” Bayesian borrowing method is considered in which the adult information is taken into account only if the observed current and external response rates coincide exactly, that is, ${\hat{p}}_{\text{ped}}={\hat{p}}_{\text{adu}}$. For the sake of argument, we assume a much larger adult trial resulting in external information of $d_{0}^{\prime }=\left\{30;100\right\}$. The response rates of external and pediatric trial coincide for ${\hat{p}}_{\text{ped}}=.3$, that is, for $r_{\text{ped}}=12$. The posterior probability with and without borrowing coincide for $r_{\text{ped}}\neq 12$, whereas the value is considerably increased with borrowing for $r_{\text{ped}}=12$, see Table 1. With extreme borrowing from adults, the threshold c=.95 corresponds to a rejection region $C_{d_{0}}=\left\{12,\ldots ,40\right\}$ and type I error of 8.8%. The threshold can, however, be selected as $c^{\prime }=.9976$. This leads to a rejection region $C_{d_{0}}=\left\{12\right\}\cup \left\{16,\ldots ,40\right\}$ with type I error 4.7%≤5%. With this rejection region, however, the power of this test is much reduced. In the terminology of Section 2.2, this is an example of a nonmonotone function $g_{d_{0}}$ and a situation in which no $c_{d_{0}}$ can be identified such that $\varphi_{B}$ and $\varphi_{\text{UMP}}$ coincide. Obviously, such a rejection region would be inacceptable in the clinical context, as a result of $r_{\text{ped}}=12$ would result in claiming efficacy of the treatment and more pediatric responders, for example, $r_{\text{ped}}=13$, would indicate inefficacy.

## 5 CONCLUSIONS AND DISCUSSION

For scenarios in which a UMP‐ or UMP‐unbiased test exists, we have shown in general that borrowing from external information cannot improve power while controlling type I error, even when borrowing is adapted to prior‐data conflict. For any general setting, the reason for this is that when external information is available, it is not random but is used as fixed information. The rejection region of the test decision rule is modified to adapt for the external information and the test is performed on basis of the (random) current data *d*_1_. We have exemplified this general statement in a setting where a one‐sample one‐sided test for a dichotomous endpoint is performed. Different borrowing approaches lead to an increase in type I error when the original Bayesian decision rule $P(p>p_{0}|\text{Data})>c$ was applied. For “reasonable” Bayesian borrowing methods such as the power prior, the robust mixture prior, and the hierarchical model approach, modification of the threshold *c* remedied the type I error inflation but converted the Bayesian decision rule to the UMP test and hence no power was gained. For the frequentist two‐stage test‐then‐pool approach, type I error was inflated as well and could be remedied by selecting a more stringent significance level for the pooled analysis. In an artificial extreme borrowing method, it was shown that the threshold *c* can be modified to control type I error but that this leads to a power decrease. Note that our argument was based on converting the decision rule used for borrowing external information to a decision rule in terms of rejection region for the current data. We argue that application of this approach provides additional insights into the frequentist operating characteristics of the borrowing method under investigation.

In the selected exemplary situation, the external adult information was chosen to be in the alternative but close to the null hypothesis region to illustrate the effect of type I error inflation most strikingly. If the external adult information is more extreme, that is, “far” in the null hypothesis or “far” in the alternative, the effect of type I error inflation will be less obvious, at least for dynamic borrowing, but in these cases, it is evident that no power can be gained because current data carry enough information by themselves and lead to acceptance or rejection of *H*_0_ without external borrowing.

Section 3 listed situations in which UMP‐ or UMP‐unbiased tests exist. Hence, our finding not only holds for the situation of one‐arm trials, but it is also true for two‐arm trials. In the case of two arms, borrowing of external information can be to one or to both arms, and it can include external information from any source, including several historical trials, for example, using a meta‐analytic predictive (MAP) prior (Neuenschwander, Capkun‐Niggli, Branson, & Spiegelhalter, 2010) or power prior (Gravestock & Held, 2019). When a UMP‐unbiased test but not a UMP test exists, for example, the two‐sided test situations mentioned in Section 3, borrowing of external information either leads to type I error inflation or to a biased test, as illustrated in the Appendix.

We believe that the present work provides a closure to the discussion, which has received increasing attention in the last few years as documented by several simulation studies, on whether adaptive borrowing mechanisms can offer any advantages in terms of error rates when UMP tests exist. We have proven that approaches adaptively discounting prior information do not offer any advantage over a fixed amount of borrowing, or no borrowing at all. It can be argued that the shape of the power curve is always the same, including the trade‐off between type I and type II error: If there is little type I error inflation, there is little power gain; for large power gain, we have to be comfortable with a possible large type I error inflation. In any case, the maximal power gain is determined by the UMP test corresponding to the inflated type I error. There exists also a notion by which prior information can be equated to a certain number of samples (the prior effective sample size), but, again, as long as prior information is conditioned upon, such samples cannot contribute to a simultaneous improvement of type I error and power.

Should then borrowing completely be discouraged? Certainly not. We just have to give up the desire for strict type I error control. In the FDA's recent draft guidance about the use of adaptive designs for clinical trials of drugs and biologics (see FDA, 2018), the concept of Bayesian adaptive designs is discussed. It is clarified that “any clinical trial whose design is governed by type I error probability and power considerations is inherently a frequentist trial.” They acknowledge that “controlling type I error at a conventional level in cases where formal borrowing is used generally limits or completely eliminates the benefits of borrowing.” Still, the FDA does not prohibit designs that borrow information from external sources but encourages discussion with its review division at an early stage, hence knowingly allowing for type I error inflation. Similar statements are given in FDA and CDRH (2010), see also Campbell (2013) for an insightful paper on FDA's regulatory view on Bayesian designs of clinical trials. Examples exist where use of external data for confirmatory trials is explicitly accepted by FDA, even though type I error rate can increase to 100%. French, Wang, Warnock, and Temkin (2017) report an analysis of epilepsy therapy studies. The specific setting in this medical area necessitates the use of data as historical control for monotherapy approval studies, and FDA accepted the concept of historical controls in this setting. Another example is the FDA guideline for noninferiority (NI) trials (see FDA, 2016), where they state that “In the absence of a placebo arm, knowing whether the trial had assay sensitivity relies heavily on external (not within‐study) information, giving NI studies some of the characteristics of a historically controlled trial.” While the European Medicines Agency (EMA), for example, EMA CHMP (2018), shows openness to Bayesian methods, it does not explicitly give guidance on how to deal with type I error inflation.

Type I error inflation can indeed be motivated by recognizing that type I and type II errors may have different implicit costs in different situations. Due to their intrinsic trade‐off, approaches have been proposed to define an optimal type I error value based on the relative importance of each (see Grieve, 2015, and references therein). The discussion is naturally linked to the fully Bayesian approach, where the parameter generating the data is considered, in turn, a random variable to which a prior distribution is assigned. In this framework, decision‐theoretic approaches can be adopted to define an optimal threshold *c* for rejection, which is associated both with the relative costs of each error, and the prior distribution that is assumed to generate the data. Note that the latter may or may not coincide with the prior distribution adopted to fit the data (see, e.g., O'Hagan, Stevens, & Campbell, 2005; Psioda & Ibrahim, 2018; Sahu & Smith, 2006; Wang & Gelfand, 2002). The prior distribution generating the data can, for example, convey the external information, and may be truncated to provide a prior distribution under the null and alternative hypothesis, as proposed in, for example, Psioda and Ibrahim (2018) for a specific borrowing situation. Integration of type I error and power with respect to a prior under the null and under the alternative hypothesis leads to the definition of a Bayesian expected type I error and power. Averaging across a set of values that include the frequentist “worst‐case” scenario leads to an average type I error that can be lower or equal to the conditional counterpart; at the same time, averaging across a set of values that include the most likely effect will generally lead to a power lower than the conditional counterpart. The authors show how such information can also be used to inform the choice of an optimal sample size. Note that Bayesian expected power or “assurance” is often regarded as a more realistic estimation of the probability of trial success Crisp, Miller, Thompson, and Best (2018); Spiegelhalter, Abrams, and Myles (2004).

Finally, an additional advantage of borrowing can be related to the fact that, although the design of a trial explores a wide range of possible outcomes, data are in reality generated by only one “true” parameter value, or prior distribution if we adopt the fully Bayesian point of view. If prior information is reliable and consistent with the new data generating process, the final trial decision *will* be associated with a lower error. We could argue that the key question should rather be if prior information is to be trusted, rather than if borrowing is beneficial for any possible true parameter value.

To summarize, we want to emphasize that borrowing is an extremely useful concept when it allows to move closer to the “true” data‐generating process. It can provide significant gains by reducing the chance of an incorrect final decision once data have been observed, and it can guide in the selection of designs that rely less on pessimistic or somewhat arbitrary (i.e., lacking to account for uncertainty) choices, such as the values at which maximum type I error and power are evaluated.

## CONFLICT OF INTEREST

The authors have declared no conflict of interest.

## Supporting information

Supporting Information

## Figures and Tables

**Figure A1:**
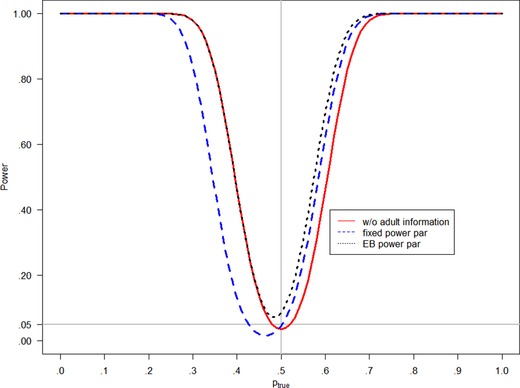
Power functions for the two‐sided test $H_{0}:p_{\text{ped}}=.5$ against the alternative $H_{1}:p_{\text{ped}}\neq .5$ without adult information, and with $r_{\text{adu}}=57$ responders among $n_{\text{adu}}=100$ adults in a fixed power parameter and an EB power parameter approach

## APPENDIX 1 BORROWING OF EXTERNAL INFORMATION IN THE TWO‐SIDED TEST SITUATION

We again consider a one‐arm trial with dichotomous endpoint and test $H_{0}:p_{\text{ped}}=p_{0}$ against the alternative $H_{1}:p_{\text{ped}}\neq p_{0}$, controlling the significance level by α=.05. We consider a slightly different scenario, assuming $n_{\text{ped}}=100$ and $p_{0}=.5$. For simplicity, we focus in the two‐sided setting on the acceptance region $\bar{C}$ rather than the critical region as in the main text.

In the stand‐alone design, the acceptance region of the UMP‐unbiased test is given by $\bar{C}=\left\{40,\ldots ,60\right\}$. In a Bayesian approach, this is obtained by accepting *H*_0_ whenever $P\left(p_{\text{ped}}>p_{0}|r_{\text{ped}},n_{\text{ped}}\right)\in \left[\;c_{1},c_{2}\right]$, with $c_{1}=.022$ and $c_{2}=1-c_{1}$. The resulting power function is given in Figure A1.

Assume that the adult trial had size $n_{\text{adu}}=100$ with $r_{\text{adu}}=57$ responders. If adult data are incorporated by a Bayesian approach with *c*_1_ and *c*_2_ as above for the test decision, use of a fixed power parameter δ=.5 leads to a shifted acceptance region $\bar{C}=\left\{35,\ldots ,58\right\}$. The respective power function in Figure A1 shows that type I error is controlled and power is increased for $p_{\text{true}}>.5$. However, incorporation of the external data results in a biased test and power is decreased for $p_{\text{true}}<.5$. Using an EB power prior approach leads to the acceptance region $\bar{C}=\left\{40,\ldots ,58\right\}$ such that type I error rate is inflated and power is increased for $p_{\text{true}}>.5$ as well as for $p_{\text{true}}$ smaller but close to .5, as shown in Figure A1.
