# Supplementary material for: Power gains by using external information in clinical trials are typically not possible when requiring strict type I error control
Source: Biom J. 2019 Jul 2;62(2):361–74. doi: 10.1002/bimj.201800395 (PMC7079072; doi:10.1002/bimj.201800395)
Supplement: Supplementary file 1 — Supporting Information [file BIMJ-62-361-s001.zip › Code/reproduce_results.pdf]

Power gains by using external information in clinical trials are typically not possible when requiring strict type I error control

Annette Kopp-Schneider, Silvia Calderazzo, Manuel Wiesenfarth

Parameters

First we set the parameters as in the paper. Notation is identical as in the paper.

```
p0=0.2
s1=0.5
s2=0.5
n.ped=40
c=0.95

n.adu=40
x.adu=12

d=0.5
library(latex2exp)
```

Planning the pediatric arm with stand-alone evaluation

Determine UMP test and check that

$$\varphi_{\text{UMP}}(r_{\text{ped}}) = \begin{cases} 1 & \text{if } r_{\text{ped}} > 12, \text{ or, equivalently, } r_{\text{ped}} \geq 13 \\ 0 & \text{if } r_{\text{ped}} \leq 12 \end{cases} \tag{1}$$

is the correct test.

```
pbinom(size=40,q=12 ,p=0.2, lower.tail=FALSE)
```

```
## [1] 0.04324162
```

Check Bayesian version:

$$P(p_{\text{ped}} > p_0 | r_{\text{ped}}, n_{\text{ped}}) \geq c. \tag{2}$$

```
postprob.woA=rep(NA,n.ped+1)
for (my.i in 1:(n.ped+1)){
  postprob.woA[my.i]=pbeta(p0,shape1=s1+my.i-1,shape2=s2+n.ped-my.i+1, lower.tail=F)
}

sum(sapply(which(postprob.woA>0.95)-1,function(x) dbinom(x,n.ped,0.2) ))
```

```
## [1] 0.04324162
```

Figure 1

Posterior probability $P(p_{\text{ped}} > p_0 | r_{\text{ped}}, n_{\text{ped}})$ as a function of the number of responders r_{ped}.

```
plot(0:40, (0:40)/40, type="n",xlab=expression(r[ped]),
     ylab=TeX("$P(p_{ped} > p_0 | r_{ped},n_{ped}=40)$"),
     xaxt="n",yaxt="n",
     main="")
axis(side=1,at=(0:40),labels=FALSE)
text(x=c(0:8)*5,par("usr")[3]-0.05 ,labels = c(0:8)*5,xpd = TRUE)
axis(side=2,at=(0:5)/5,las=1)
abline(v=13,lty=2,col="grey",lwd=1)
lines((0:40),postprob.woA,
      type="l",col="grey",lwd=1)
points((0:12),postprob.woA[1:13],
       col="red",pch=15,cex=1.2)
points((13:40),postprob.woA[14:41],
       col="green",pch=17,cex=1.2)
abline(h=0.95,lty=1,col="black",lwd=1.5)
text(x=35,y=0.92,labels="c = 0.95")
```

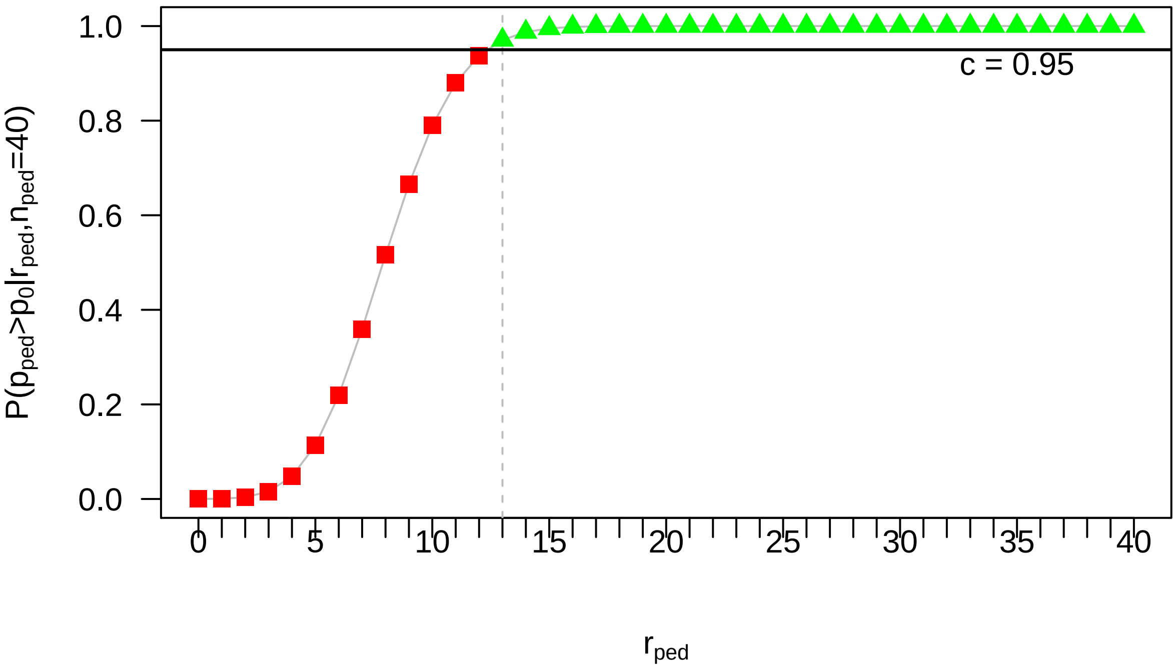

Figure 2

All possible power functions for UMP-tests in the situation $n_{\text{ped}} = 40$, varying the threshold b for the observed number of responders, or equivalently the threshold c for the posterior probability. The values of $p_{\text{true}} = 0.2$

and $\alpha = 0.05$ are indicated as vertical and horizontal grey lines and the power curve for $b = 13$, corresponding to the 5% level UMP test, is indicated in dashed red.

```
plot(0:40/40, (0:40)/40, type="n",xlab=expression(p[true]),ylab="Power",xaxt="n",yaxt="n",
     main="")
axis(side=1,at=(0:10)/10)
axis(side=2,at=c(0,0.05,0.2,0.4,0.6,0.8,1),las=1)
for (my.i in c(-1:11,13:39)){
  lines((0:100)/100,sapply((0:100)/100, function(cur) pbinom(size=40,q=my.i ,p=cur, lower.tail=FALSE)),
        type="l",col=1,lwd=2)
}
lines((0:100)/100,sapply((0:100)/100, function(cur) pbinom(size=40,q=12 ,p=cur, lower.tail=FALSE)),
      type="l",col=2,lty="longdash",lwd=3)
abline(h=0.05,col="grey",lwd=2)
abline(v=0.2,col="grey",lwd=2)
```

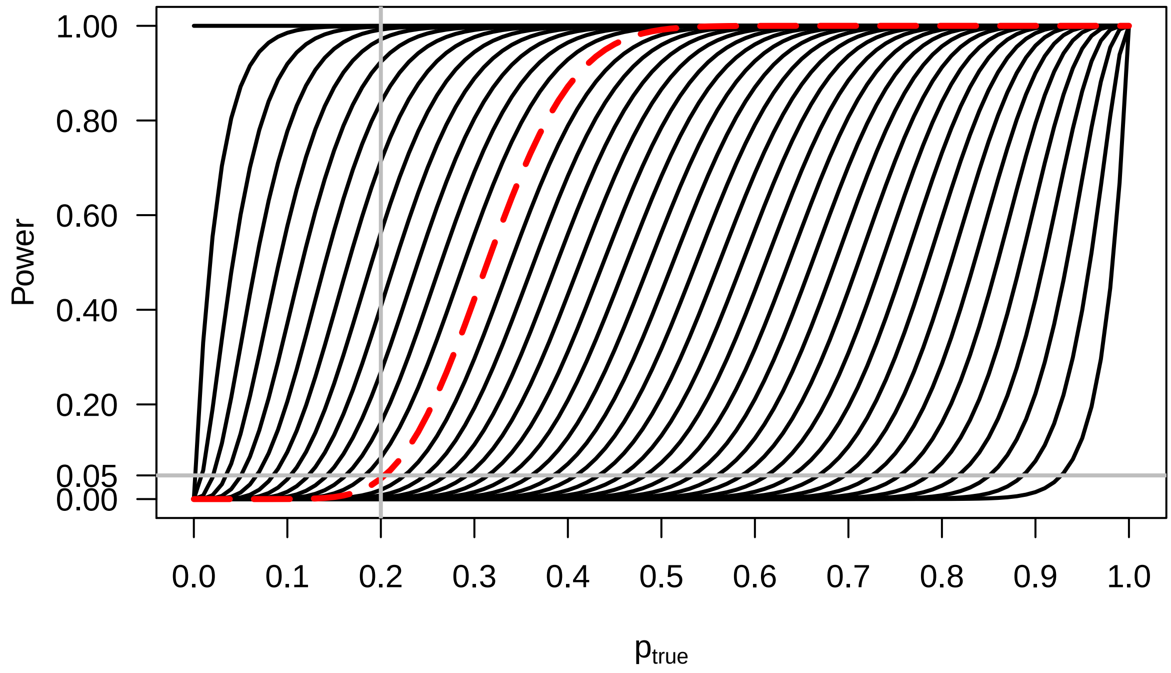

Planning the pediatric arm with borrowing from external information

Borrowing from the adult trial using the power prior approach:

Fixed power parameter

Determine $\mathcal{C}_{d_0}$ for $c = 0.95$ and for $c_{d_0} = 0.97$:

```
postprob.wAfixedd=rep(NA,n.ped+1)
for (my.i in 1:(n.ped+1)){
  postprob.wAfixedd[my.i]=pbeta(p0,shape1=s1+d*x.adu+my.i-1,
                                shape2=s2+d*(n.adu-x.adu)+n.ped-my.i+1, lower.tail=F)
}

which(postprob.wAfixedd>0.95)-1
```

```
##  [1] 12 13 14 15 16 17 18 19 20 21 22 23 24 25 26 27 28 29 30 31 32 33 34
## [24] 35 36 37 38 39 40
```

```
which(postprob.wAfixedd>0.97)-1
```

```
##  [1] 13 14 15 16 17 18 19 20 21 22 23 24 25 26 27 28 29 30 31 32 33 34 35
## [24] 36 37 38 39 40
```

Adaptive power parameter: Empirical Bayes approach

EB deltas are generated by function EB_fullAdults.

```
library(StudyPrior)
library(edfun)

##################################################
##only use powerpar from binom.PP.EB.
 EB_fullAdults=function(current.n,hist.n,shape1E,shape2E){
   Xvec=0:current.n
   prior=lapply((0:hist.n), function(x) binom.PP.EB(x,hist.n,Xvec,current.n,p.prior.a =shape1E,p.prior.[illegible]
  powerpar=matrix(NA,nrow=hist.n+1,ncol=current.n+1,dimnames=list(paste0("hist",0:hist.n),paste0("cur",[illegible]
  for(x in (1:(hist.n+1))){
    for(X in 0:current.n){
      powerpar[x,X+1]=   environment(prior[[x]])$ds[[X+1]]
    }
  }
  list(powerpar=powerpar)
}

EB.n40_40_fullAdults=EB_fullAdults(current.n=40,hist.n=40,shape1E=0.5,shape2E=0.5)
save(list =ls()[grep("EB",ls())],file= "EB40_40.RData")
```

Since this is time consuming, deltas are loaded from workspace.

```
load("EB40_40.RData")

s1_wA=s1 + x.adu*EB.n40_40_fullAdults$powerpar["hist12",]
s2_wA=s2 + (n.adu-x.adu)*EB.n40_40_fullAdults$powerpar["hist12",]


postprob.wAEB=rep(NA,n.ped+1)
for (my.i in 1:(n.ped+1)){
  postprob.wAEB[my.i]=pbeta(p0,shape1=s1_wA[my.i]+my.i-1,shape2=s2_wA[my.i]+n.ped-my.i+1, lower.tail=F)
}
```

Mixture prior approach

The prior mixture weight is set to 0.5. We use a mixture between Jeffrey's prior and the full adult information.

```
weight=0.5

d=1
a1=s1 + d*x.adu
b1=s2+d*(n.adu-x.adu)

a2=b2=s1

weightMP=function(weight,shapeuninf1,shapeuninf2,x.ped,n.ped,x.adu,n.adu) {
  shapeinf1=shapeuninf1+x.adu
  shapeinf2=shapeuninf2+(n.adu-x.adu)

  const1=weight*beta(x.ped+shapeinf1,n.ped-x.ped+shapeinf2)/beta(shapeinf1,shapeinf2)
  const2=(1-weight)*beta(x.ped+shapeuninf1,n.ped-x.ped+shapeuninf2)/beta(shapeuninf1,shapeuninf2)
  const=const1+const2

  out=weight*beta(x.ped+shapeinf1,n.ped-x.ped+shapeinf2)/beta(shapeinf1,shapeinf2)/const
}

postprob.wAMP=rep(NA,n.ped+1)
wMP=rep(NA,n.ped+1)

for (my.i in 1:(n.ped+1)){
  wMP[my.i]=weightMP(weight,shapeuninf1=a2,shapeuninf2=b2,my.i-1,n.ped,x.adu=x.adu,n.adu=n.adu)

  postprob.wAMP[my.i]= wMP[my.i]*pbeta(p0,shape1=a1+my.i-1,shape2=b1+n.ped-my.i+1, lower.tail=F)+
    (1-wMP[my.i])*pbeta(p0,shape1=a2+my.i-1,shape2=b2+n.ped-my.i+1, lower.tail=F)
}
```

Figure 3

Adaptive power parameter $\hat{\delta}$ determined by Empirical Bayes and posterior weight of the robust mixture prior. Results are given for $n_{\text{ped}} = 40$, adult information $d_0 = \{r_{\text{adu}} = 12; n_{\text{adu}} = 40\}$ and prior weight $w = 0.5$ for the robust mixture prior approach.

```
plot(0:40, (0:40)/40, type="n",xlab=expression(r[ped]),
     ylab="",
     xaxt="n",yaxt="n",
     main="")

axis(side=1,at=(0:40),labels=FALSE)
text(x=c(0:8)*5,par("usr")[3]-0.05 ,labels = c(0:8)*5,xpd = TRUE)
axis(side=2,at=(0:5)/5,las=1)
axis(side=4,at=(0:5)/5,las=1)
mtext(side=2,text=TeX("EB Estimated power parameter,  $\\hat{\\delta}$"),line=3)
mtext(side=4,text=TeX("posterior weight, $\\tilde{w}$"),line=3)

lines((0:40),EB.n40_40_fullAdults$powerpar["hist12",][1:41],
      type="l",col="grey",lty=1,lwd=1)
```

```
points((0:40),EB.n40_40_fullAdults$powerpar["hist12",][1:41],
       col="red",pch=4,cex=1.2,lwd=2)

lines((0:40),wMP[1:41],
      type="l",col="grey",lwd=1)
points((0:40),wMP[1:41],
       col="blue",pch=3,cex=1.2,lwd=2)

legend("topright",legend=c("EB power parameter","Mixture prior weight"),
       col=c("red","blue"),pch=c(4,3))
```

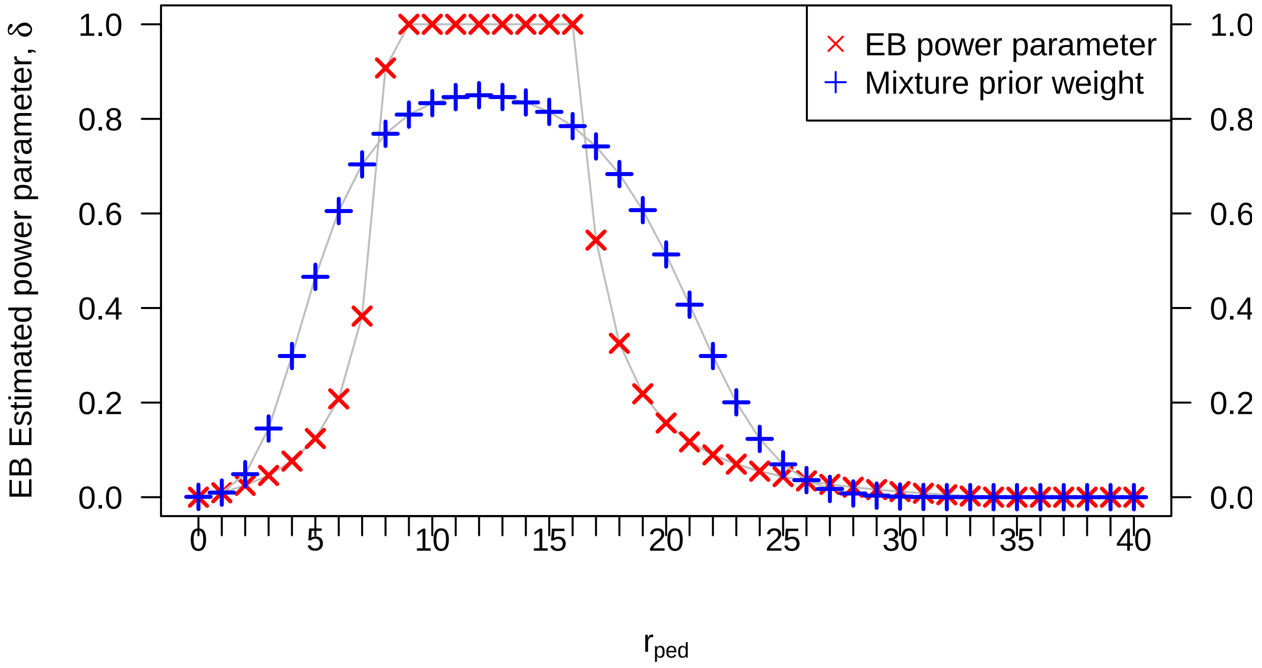

Hierarchical model

The code for evaluating the posterior probabilities is given by:

```
stanmodelcode <- "
data {
  int<lower=0> groups;
  int<lower=0> length_y;
  int<lower=0> y[length_y];
  int<lower=0> n;
  int<lower=0> n_nuisance;
  int<lower=0> r_nuisance;
  real mu_mean;
```

```
  real<lower=0> mu_sd;
  real<lower=0> hyper_sd;
}

transformed data {
  int<lower=0> r_nuisance_mat[length_y];
  int<lower=0> r_mat[length_y,2];
  int<lower=0> nvec[2];
  for (y_i in 1:length_y)
    r_nuisance_mat[y_i]=r_nuisance;
  r_mat[,1]=y;
  r_mat[,2]=r_nuisance_mat;
  nvec[1]=n;
  nvec[2]=n_nuisance;
}

parameters {
  row_vector[groups] LogOdds_raw[length_y];
  real mu[length_y];
  real<lower=0> tau[length_y];
}

transformed parameters {
  row_vector[groups] LogOdds[length_y];
  row_vector[groups] pp[length_y];
  real p[length_y];
  for (y_i in 1:length_y){
    LogOdds[y_i]=mu[y_i]+tau[y_i]* LogOdds_raw[y_i];
    pp[y_i]=inv_logit(LogOdds[y_i]);
  }
  p=pp[,1];
}

model {
  for (y_i in 1:length_y){
    mu[y_i]~normal(mu_mean,mu_sd);
    tau[y_i] ~ normal(0,hyper_sd);
    LogOdds_raw[y_i]~normal(0,1);
    r_mat[y_i]~binomial_logit(nvec,LogOdds[y_i]);
  }
}
"


library(rstan)
mod=stan_model(model_code =stanmodelcode)
y=0:40
k=40
set.seed(1)
ddd=c(list(y=y,n=k,length_y=length(y)),
      list(mu_mean=0,mu_sd=2,hyper_sd=1,groups=2,r_nuisance=as.integer(12),n_nuisance=as.integer(40)))
fit=rstan::sampling(mod,
                    data=ddd,
```

```
                    iter=100000,warmup=5000,chains=4,cores=1,
                    algorithm="NUTS",
                    verbose = F,open_progress=T
)
pmat=extract(fit,pars="p")
pprob=colSums(apply(pmat$p,2,function(z) z>0.2))/nrow(pmat$p)
rm(fit)
save.image(file="d:/hierarchUMP_revised_small.RData")
```

Since this is time consuming, posterior probabilities are loaded from workspace.

```
load("hierarchUMP_revised_small.RData")

postprob.wAHM=pprob
```

Figure 4

Posterior probability $P(p_{\text{ped}} > p_0|\text{Data})$ as a function of the number of responders r_{ped} without external information, and with adult information $d_0 = \{r_{\text{adu}} = 12, n_{\text{adu}} = 40\}$, using a fixed power parameter ($\delta = 0.5$), the EB power parameter, a mixture prior approach with $w = 0.5$ and a hierarchical model. The posterior probability for extreme borrowing follows the one without external information, except for $r_{\text{ped}} = 12$, where it jumps to 0.9977.

```
plot(0:18, (0:18)/18, type="n",xlab=expression(r[ped]),
     ylab=TeX("$P(p_{ped} > p_0 | r_{ped},n_{ped}=40;r_{adu}=12,n_{adu}=40)$"),
     xaxt="n",yaxt="n",
     main="")

axis(side=1,at=(0:18))
axis(side=2,at=(0:5)/5,las=1)

lines((0:18),postprob.woA[1:19],
      type="l",col="grey",lty=1,lwd=1)
points((0:18),postprob.woA[1:19],
       col="red",pch=1,cex=1.2,lwd=2)

lines((0:18),postprob.wAfixedd[1:19],
      type="l",col="grey",lwd=1)
points((0:18),postprob.wAfixedd[1:19],
       col="red",pch=3,cex=1.2,lwd=2)

lines((0:18),postprob.wAEB[1:19],
      type="l",col="grey",lwd=1)
points((0:18),postprob.wAEB[1:19],
       col="green",pch=4,cex=1.2,lwd=2)

lines((0:18),postprob.wAMP[1:19],
      type="l",col="grey",lwd=1)
points((0:18),postprob.wAMP[1:19],
       col="black",pch=4,cex=1.2,lwd=2)

lines((2:18),postprob.wAHM[3:19],
      type="l",col="grey",lwd=1)
```

```r
points((2:18),postprob.wAHM[3:19],
       col="blue",pch=4,cex=1.2,lwd=2)

abline(h=0.95,lty=1,col="black",lwd=1.5)
text(x=16,y=0.92,labels="c = 0.95")

legend("bottomright",legend=c("w/o adult information","fixed power par","EB power par","Mixture prior",
       col=c("red","red","green","black","blue"),pch=c(1,3,4,4,4))
```

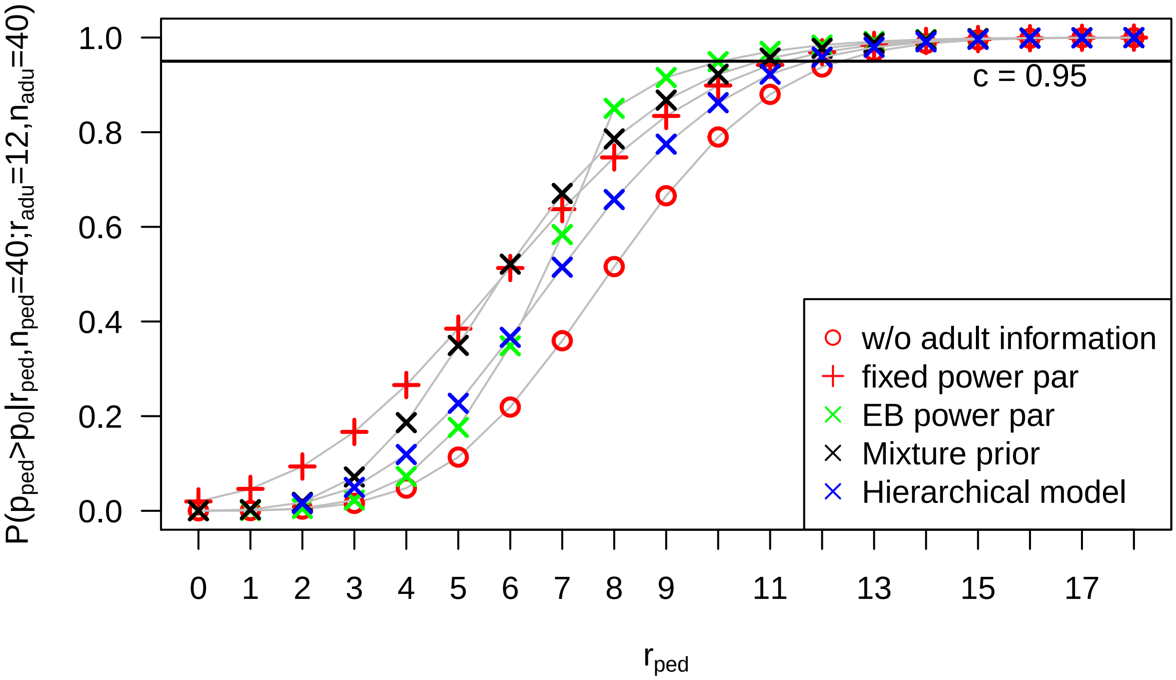

Extreme borrowing

Adults with 30 out of 100 responses are borrowed completely when rates coincide, i.e., when 12 out of 40 peds. Determine $\mathcal{C}_{d_0}$ for $c = 0.95$ and for $c_{d_0} = 0.9976$:

```r
unitVector=function(i,n){
  a=rep(0,n+1)
  a[i+1]=1
  a
}

s1_wA_30=s1 + unitVector(12,40)*30
s2_wA_30=s2 + unitVector(12,40)*(100-30)

postprob.wAExB_30=rep(NA,40+1)
```

```
for (my.i in 1:(40+1)){
  postprob.wAExB_30[my.i]=pbeta(p0,shape1=s1_wA_30[my.i]+my.i-1,shape2=s2_wA_30[my.i]+40-my.i+1, lower.
}

which(postprob.wAExB_30>0.99765)-1
```

```
##  [1] 12 16 17 18 19 20 21 22 23 24 25 26 27 28 29 30 31 32 33 34 35 36 37
## [24] 38 39 40
```

```
sum(sapply(which(postprob.wAExB_30>0.9976)-1,function(x) dbinom(x,n.ped,0.2) ))
```

```
## [1] 0.04719981
```

```
which(postprob.wAExB_30>0.95)-1
```

```
##  [1] 12 13 14 15 16 17 18 19 20 21 22 23 24 25 26 27 28 29 30 31 32 33 34
## [24] 35 36 37 38 39 40
```

```
sum(sapply(which(postprob.wAExB_30>0.95)-1,function(x) dbinom(x,n.ped,0.2) ))
```

```
## [1] 0.08750524
```

Table 1 entries

```
postprob.woA[10:17]
```

```
## [1] 0.6657403 0.7898335 0.8799489 0.9377403 0.9706813 0.9874569 0.9951216
## [8] 0.9982740
```

```
postprob.wAfixedd[10:17]
```

```
## [1] 0.8343675 0.8987070 0.9420874 0.9690452 0.9845278 0.9927653 0.9968338
## [8] 0.9987025
```

```
postprob.wAEB[10:17]
```

```
## [1] 0.9155562 0.9490428 0.9708091 0.9841236 0.9917999 0.9959766 0.9981241
## [8] 0.9991686
```

```
postprob.wAMP[10:17]
```

```
## [1] 0.8678480 0.9225018 0.9568206 0.9771639 0.9885515 0.9945690 0.9975682
## [8] 0.9989759
```

```
postprob.wAHM[10:17]
```

```
## [1] 0.7747605 0.8623895 0.9225211 0.9585421 0.9795263 0.9909974 0.9961421
## [8] 0.9985632
```

```
postprob.wAExB_30[10:17]
```

```
## [1] 0.6657403 0.7898335 0.8799489 0.9976500 0.9706813 0.9874569 0.9951216
## [8] 0.9982740
```

Test then pool

```
p.threshold=0.05
res1=rep(NA,n.ped) #first-stage test: pooled evaluation?
res2=rep(NA,n.ped) # second-stage test

for (my.i in (1:n.ped)){
  x=matrix(c(my.i,12,n.ped-my.i,28),nrow=2,ncol=2)
  y=fisher.test(x,alternative = "two.sided")
  res1[my.i]= y$p.value
  if (res1[my.i] > p.threshold) {
    yy=binom.test(my.i+12,n.ped+40,p=0.2,alternative="greater")
    res2[my.i]=yy$p.value
  } else {
    yy=binom.test(my.i,n.ped,p=0.2,alternative="greater")
    res2[my.i]=yy$p.value
  }
}
```

pooled analysis for

```
which(res1>0.2)
```

```
##  [1]  7  8  9 10 11 12 13 14 15 16 17 18
```

Type I error inflation for $\alpha_{d_0} = 5\%$:

```
which(res2<0.05)
```

```
##  [1] 11 12 13 14 15 16 17 18 19 20 21 22 23 24 25 26 27 28 29 30 31 32 33
## [24] 34 35 36 37 38 39 40
```

is corrected by setting $\alpha_{d_0} = 2\%$:

```
which(res2<0.02)
```

```
##  [1] 13 14 15 16 17 18 19 20 21 22 23 24 25 26 27 28 29 30 31 32 33 34 35
## [24] 36 37 38 39 40
```

Appendix

Figure 5

Power functions for the two-sided test $H_0 : p_{\text{ped}} = 0.5$ against the alternative $H_1 : p_{\text{ped}} \neq 0.5$ without adult information, and with $r_{\text{adu}} = 57$ responders among $n_{\text{adu}} = 100$ adults in a fixed power parameter and an EB power parameter approach.

```
powertwosided=function(acclower,accupper,n,p){
  xx=pbinom(size=n,q=acclower-1 ,p=p, lower.tail=TRUE)+pbinom(size=n,q=accupper ,p=p, lower.tail=FALSE)
  return(xx)
}

#########without adults
n.ped_Fig5=100
p0_Fig5=0.5
```

```
pbinom(size=n.ped_Fig5,q=60 ,p=p0_Fig5, lower.tail=FALSE) #P(X>60)
```

```
## [1] 0.0176001
```

```
#[1] 0.0176001
pbinom(size=n.ped_Fig5,q=39,p=p0_Fig5, lower.tail=TRUE) #P(X<= 39)
```

```
## [1] 0.0176001
```

```
#[1] 0.0176001
###acceptance region for 5% level test: 40 <= x <=60
powertwosided(acclower=40,accupper=60,n=n.ped_Fig5,p=p0_Fig5) #[1] 0.0352002
```

```
## [1] 0.0352002
```

```
postprob.woA_Fig5=rep(NA,n.ped_Fig5+1)
for (my.i in 1:(n.ped_Fig5+1)){
  postprob.woA_Fig5[my.i]=pbeta(p0_Fig5,shape1=s1+my.i-1,shape2=s2+n.ped_Fig5-my.i+1, lower.tail=FALSE)
}
#which(postprob.woA >= 0.025 & postprob.woA <= 0.975)
which(postprob.woA_Fig5 >= 0.022 & postprob.woA_Fig5 <= 0.978)-1
```

```
##  [1] 40 41 42 43 44 45 46 47 48 49 50 51 52 53 54 55 56 57 58 59 60
```

```
# [1] 40 41 42 43 44 45 46 47 48 49 50 51 52 53 54 55 56 57 58 59 60

#########with adults, fixed power parameter
n.adu_Fig5=100
x.adu_Fig5=57

d=0.5
postprob.wAfixedd_Fig5=rep(NA,n.ped_Fig5+1)
for (my.i in 1:(n.ped_Fig5+1)){
  postprob.wAfixedd_Fig5[my.i]=pbeta(p0_Fig5,shape1=s1+d*x.adu_Fig5+my.i-1,
                                shape2=s2+d*(n.adu_Fig5-x.adu_Fig5)+n.ped_Fig5-my.i+1, lower.tail=F)
}

which(postprob.wAfixedd_Fig5 >= 0.022 & postprob.wAfixedd_Fig5 <= 0.978)-1
```

```
##  [1] 35 36 37 38 39 40 41 42 43 44 45 46 47 48 49 50 51 52 53 54 55 56 57
## [24] 58
```

```
#[1] 35 36 37 38 39 40 41 42 43 44 45 46 47 48 49 50 51 52 53 54 55 56 57 58


########## EB deltas generated by generateEB.R
#EB.n100_100_fullAdults=EB_fullAdults(current.n=100,hist.n=100,shape1E=0.5,shape2E=0.5)
#save(list =ls()[grep("EB",ls())],file= "EB.n100_100_fullAdults.RData")
load("EB.n100_100_fullAdults.RData")

##delta from fullAdults, but downscaled adults enter the posterior
shape1E_wA=s1 + x.adu_Fig5*EB.n100_100_fullAdults$powerpar["hist57",]
shape2E_wA=s2 + (n.adu_Fig5-x.adu_Fig5)*EB.n100_100_fullAdults$powerpar["hist57",]

postprob.wAEB_Fig5=rep(NA,n.ped_Fig5+1)
for (my.i in 1:(n.ped_Fig5+1)){
  postprob.wAEB_Fig5[my.i]=pbeta(p0_Fig5,shape1=shape1E_wA[my.i]+my.i-1,shape2=shape2E_wA[my.i]+n.ped_Fi
```

```
}
which(postprob.wAEB_Fig5 >= 0.022 & postprob.wAEB_Fig5 <= 0.978)-1
```

```
##  [1] 40 41 42 43 44 45 46 47 48 49 50 51 52 53 54 55 56 57
```

```
#[1] 40 41 42 43 44 45 46 47 48 49 50 51 52 53 54 55 56 57


plot(0:100/100, (0:100)/100, type="n",xlab=expression(p[true]),ylab="Power",xaxt="n",yaxt="n",
     main="")
axis(side=1,at=(0:10)/10)
axis(side=2,at=c(0,0.05,0.2,0.4,0.6,0.8,1),las=1)
abline(h=0.05,col="grey",lwd=2)
abline(v=0.5,col="grey",lwd=2)

#w/o adults
lines((0:100)/100,sapply((0:100)/100, function(cur) powertwosided(acclower=40,accupper=60,n=100,p=cur))
      type="l",col="red",lty=1,lwd=3)

#fixed pp
lines((0:100)/100,sapply((0:100)/100, function(cur) powertwosided(acclower=35,accupper=58,n=100,p=cur))
      type="l",col="blue",lty=2,lwd=3)

#EB pp
lines((0:100)/100,sapply((0:100)/100, function(cur) powertwosided(acclower=40,accupper=57,n=100,p=cur))
      type="l",col="black",lty=3,lwd=3)
legend(x=0.61,y=0.4,legend=c("w/o adult information","fixed power par","EB power par"),
       col=c("red","blue","black"),lty=c(1:3))
```

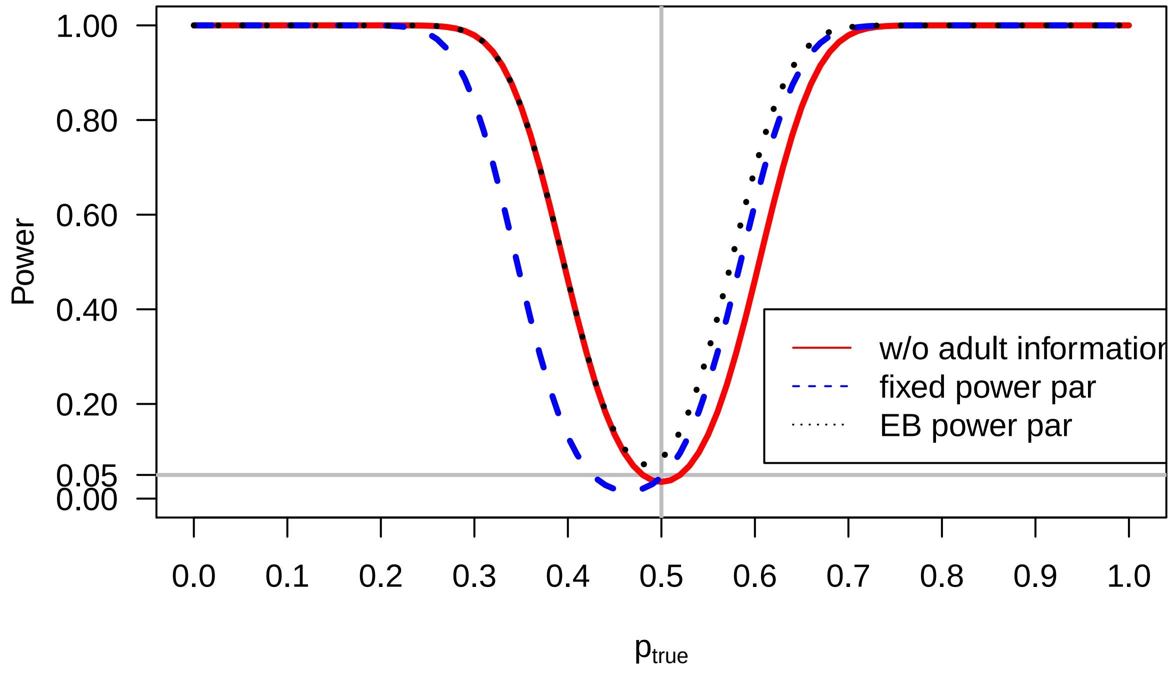

Session info

```r
sessionInfo()
```

```
## R version 3.4.3 (2017-11-30)
## Platform: x86_64-w64-mingw32/x64 (64-bit)
## Running under: Windows 10 x64 (build 14393)
##
## Matrix products: default
##
## locale:
## [1] LC_COLLATE=German_Germany.1252  LC_CTYPE=German_Germany.1252
## [3] LC_MONETARY=German_Germany.1252 LC_NUMERIC=C
## [5] LC_TIME=German_Germany.1252
##
## attached base packages:
## [1] stats     graphics  grDevices utils     datasets  methods   base
##
## other attached packages:
## [1] latex2exp_0.4.0
##
## loaded via a namespace (and not attached):
##  [1] Rcpp_0.12.16       pillar_1.2.1       compiler_3.4.3
##  [4] plyr_1.8.4         bindr_0.1.1        tools_3.4.3
##  [7] RBesT_1.3-3        digest_0.6.15      evaluate_0.10.1
```

```
## [10] tibble_1.4.2       gtable_0.2.0       checkmate_1.8.5
## [13] pkgconfig_2.0.1    rlang_0.3.1        yaml_2.1.18
## [16] mvtnorm_1.0-7      bindrcpp_0.2.2     gridExtra_2.3
## [19] stringr_1.3.0      dplyr_0.7.4        knitr_1.20
## [22] stats4_3.4.3       rprojroot_1.3-2    grid_3.4.3
## [25] glue_1.2.0         inline_0.3.14      R6_2.2.2
## [28] rmarkdown_1.9      rstan_2.17.3       Formula_1.2-2
## [31] ggplot2_3.1.0      magrittr_1.5       codetools_0.2-15
## [34] backports_1.1.2    scales_0.5.0       htmltools_0.3.6
## [37] StanHeaders_2.17.2 assertthat_0.2.0   colorspace_1.3-2
## [40] stringi_1.1.6      lazyeval_0.2.1     munsell_0.4.3
```
